# Virtual Hand Feedback Reduces Reaction Time in an Interactive Finger Reaching Task

**DOI:** 10.1371/journal.pone.0154807

**Published:** 2016-05-04

**Authors:** Johannes Brand, Marco Piccirelli, Marie-Claude Hepp-Reymond, Manfred Morari, Lars Michels, Kynan Eng

**Affiliations:** 1 Institute of Neuroinformatics, University of Zurich and ETH Zurich, Zurich, Switzerland; 2 Institute of Neuroradiology, University Hospital Zurich, Zurich, Switzerland; 3 Centre for MR-Research, University Children’s Hospital, Zurich, Switzerland; 4 Automatic Control Laboratory, ETH Zurich, Zurich, Switzerland; University of Ottawa, CANADA

## Abstract

Computer interaction via visually guided hand or finger movements is a ubiquitous part of daily computer usage in work or gaming. Surprisingly, however, little is known about the performance effects of using virtual limb representations versus simpler cursors. In this study 26 healthy right-handed adults performed cued index finger flexion-extension movements towards an on-screen target while wearing a data glove. They received each of four different types of real-time visual feedback: a simple circular cursor, a point light pattern indicating finger joint positions, a cartoon hand and a fully shaded virtual hand. We found that participants initiated the movements faster when receiving feedback in the form of a hand than when receiving circular cursor or point light feedback. This overall difference was robust for three out of four hand versus circle pairwise comparisons. The faster movement initiation for hand feedback was accompanied by a larger movement amplitude and a larger movement error. We suggest that the observed effect may be related to priming of hand information during action perception and execution affecting motor planning and execution. The results may have applications in the use of body representations in virtual reality applications.

## Introduction

Many forms of computer-mediated interaction use cursors to provide real-time visual feedback of hand position while performing a task. Real-time visual feedback of hand position is compared to sensory consequences predicted by internal movement models to control the motor task [1]. Despite the ubiquity of this type of interaction, little is known about the best type of feedback to use. Most commonly, a simple point or circle or other compact abstract symbol is used to indicate current hand position. But what happens to user performance when an extended and even articulated marker is used, for example a representation of the user’s hand? Might there be some advantages in using representations which bear some correspondence to the user’s body image?

Previous human studies found behavioural differences in goal-directed target reaching between viewing a hand at movement initiation, either real [2] or virtual [3], and viewing a circular cursor. Veilleux & Proteau [2] reported lower overall movement variability during hand feedback, while Sober & Sabes [3] found a higher initial directional error for movements with a visually displaced virtual hand compared to movements with a visually displaced cursor. According to both studies, additional joint and arm configuration information available from hand visual feedback improved initial hand configuration estimation. The studies by Veilleux & Proteau [2] and Sober & Sabes [3] used tasks in which various visual targets in different movement directions had to be accurately intercepted for task success. These tasks might have particularly benefited from initial joint configuration information, while tasks without these directional requirements might not.

Body part representations serving as movement feedback may have some advantages besides just providing joint configuration information. One indication comes from studies investigating visual processing in the brain. These studies reported specific brain regions for visual processing of the human body and body parts [4]. Thus, potentially optimized neural circuits for faster visual processing of human body part feedback could facilitate action understanding and visuomotor control.

Images of human hands can be presented in different degrees of realism. Evidence of the influence of visual feedback appearance on movements comes from several motor interference experiments. In these experiments human subjects performed arm movements while watching an agent moving congruently or incongruently to their own movements [5–7]. Kilner et al. (2003) found that observation of incongruent human movement feedback interfered with actions, while observation of incongruent robotic feedback did not [5]. Subsequent studies demonstrated that robotic agents with similar motility to humans evoked motor interference [7], suggesting that human-like motility is the important part of movement feedback. This result supports the previous findings by Veilleux & Proteau [2] and Sober & Sabes [3] by further indicating the influence of observation of human-like joint configurations on movement generation.

While it seems clear that observation of movement-relevant joint information affects motor accuracy, the effect of body part resemblance in movement feedback are still to be investigated. Can some aspects of human motor performance be facilitated by seeing virtual limbs? And what are the most important attributes of the virtual limbs that lead to these performance improvements, if they indeed exist?

The present study was designed to test the behavioural effects of different types of visual hand feedback on human movements. Participants performed simple goal-directed visually-guided finger movements under four different visual feedbacks: a fully shaded virtual hand representation; a flat cartoon-like hand representation; small circles marking the index finger joints (point light); and a circle indicating the fingertip position only. Comparisons between the feedback types allowed us to study the behavioural effects on three levels: appearance (virtual hand > cartoon hand), hand shape (cartoon hand > point light), and joint information (point light > cursor). We hypothesized that feedback with body part information compared to feedback without body part information would lead to a measurable difference in movement behaviour.

## Materials and Methods

### Participants

Twenty-seven right-handed healthy paid volunteers (8 females) participated in the study and had normal or corrected-to-normal vision [8]. One participant was excluded because of incomplete data acquisition. The remaining 26 subjects were on average 27.8 years (SD 6.8 years) old and gave written informed consent prior to participation. The study design was approved by the “Kantonale Ethikkommission Zürich” (http://www.kek.zh.ch) and the experiments were conducted in compliance with the Declaration of Helsinki.

### Technical setup

**Fig 1:**
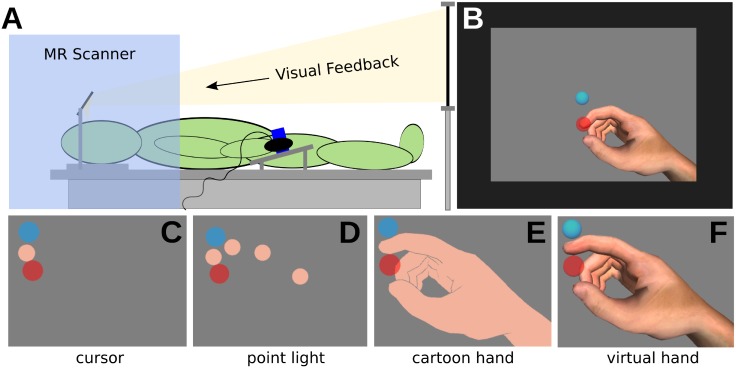
Experimental setup, visual feedback and experimental conditions. (A) Subject in the MR scanner wearing a data glove and grasping the tube with the right hand. Visual feedback provided via mirror projection from a monitor. (B) Visual feedback consisted of the starting position (light-blue circle), movement cursor (skin coloured circle) and target (red circle) on grey background. (C) cursor feedback, (D) point light feedback, (E) cartoon hand feedback, and (F) virtual hand feedback.

The experiment setup is illustrated in Fig 1. The experiment was performed with subjects lying in the bore of a magnetic resonance scanner. Functional magnetic resonance imaging data was acquired in this study; the neuroimaging results will be communicated in a follow-up publication. During the experiment subjects held a rigid plastic tube in a power grip with their right hand. The vertical tube was fixed to the bed and oriented to maintain the hand in a comfortable neutral position. This ensured that the position of the hand and fingers was approximately consistent across participants. We used tubes with three diameters (5.1, 4.7 and 4.3 cm) to adjust for varying hand sizes. We measured index finger movements with a 5DT Data Glove 5 MRI (http://www.5dt.com). Data acquisition, data processing and presentation of real-time visual feedback were done using Unity3D (version 3, http://www.unity3d.com). Task and movement feedback were displayed in real-time on a LCD monitor which participants observed via a mirror projection. Underneath the monitor an Eyelink 1000 long range video oculography system (SR-Research Ltd., ON, Canada; http://www.sr-research.com) was mounted to record the movements of one eye. The eye tracker was calibrated with a 9-point calibration routine prior to the experiment, and data was recorded at 250 Hz.

Glove sensor data was acquired at 75 Hz. The one-dimensional finger bending data was smoothed with a moving average filter over a 100 ms window. At every time point the glove input was used to infer index finger joint angles from a lookup table, which were then applied to a realistic virtual hand model. The lookup table contained joint angle mapping data that was previously acquired by motion capture (Vicon, USA) of finger flexion-extension movements. The glove was calibrated for every subject before the experiment at 100% and 0% index finger extension. The task was performed from 95% to 5% index finger extension; in this paper we report distances relative to this movement range.

For the visual feedback a realistic virtual hand model from the 5DT data glove software was used (2450 polygons). Virtual marker spheres, sized to approximately the same thickness as the virtual index fingertip, were placed on the virtual index fingertip and on the finger joint positions. Four hand feedback conditions were defined:

**Cursor (**Fig 1C**):** invisible hand model, fingertip sphere visible only, flat flesh-coloured shading, orthographic 2D projection
**Point light (**Fig 1D**):** invisible hand model, all finger spheres visible, flat flesh-coloured shading, orthographic 2D projection
**Cartoon (**Fig 1E**):** visible hand model, all spheres invisible, flat flesh-coloured shading, perspective 3D projection
**Virtual hand (**Fig 1F**):**visible hand model, all spheres invisible, realistic shading, perspective 3D projection

The visual angle of the task range on the screen was adjusted for every subject to approximately match the visual angle of the corresponding real finger movements in the lying task position, with the arm almost fully extended.

### Experimental Protocol

Subjects were instructed to operate the movements of the virtual effector (hand or circles) on the screen by extending and flexing their right index finger. They were asked to only move the index finger and to stabilize the other fingers by holding the tube. The task started with movements of the cursor into the starting position at 100% of index finger extension, represented by a blue circle (Fig 1B). After 2 s, the trial started with a red circle appearing at a pseudo-random target location (between 40–50% finger extension). The participants were instructed to immediately move the cursor as fast and accurately as possible to the target, and then immediately back to the starting position. Each trial lasted 2 s, with the target automatically disappearing after 1 s.

The trials (movements) were grouped in blocks of nine and each block lasted for 22 s (2 s task instruction, 2 s for moving the cursor into the starting position and nine trials of 2 s duration each). The blocks were interleaved with resting periods of pseudo-random length (7 to 9 s, average 8 s). The whole experiment consisted of four movement conditions, each comprising ten blocks. The conditions differed in the feedback provided during the task (Fig 1C–1F). The visual feedback was always visible during the entire blocks and a blue fixation cross was presented during the rest periods. Each condition was assigned randomly to one of four experimental runs. Each run also contained one of four randomly assigned observation conditions, in which subjects watched pre-recorded movements of the virtual effector on the screen under the four hand feedback conditions. These observation control conditions were only relevant for the analyses of eye movements and will therefore only be mentioned with respect to eye movement data in the rest of this article. Within each run, the blocks of the movement and the observation conditions were presented in random order. For 2 s before each block the word “action” (in red) or the word “observation” (in green) was presented. In between runs, participants could take a short break, if desired. The whole experiment lasted for approximately one hour, including setup time.

### Movement data analysis

We recorded the index finger movements and logged the block onsets with Unity3D. We used Matlab for basic movement analysis and feature calculation [9] and R for statistical analyses [10]. We created figures using ggplot2 [11]. The movements were automatically classified from the recorded 2 s trials by a simple algorithm using thresholds: the starting position was defined as the movement range in which the virtual finger or cursor overlapped with the starting position circle. A movement onset was then detected when 10% of the distance from the starting position to the target distance was exceeded. A movement ended as soon as the fingertip returned to the starting position (at 82% of full finger extension). All trials for which movement onset or ending could not be detected were classified as invalid. 1.0% of the movements were classified as invalid and this value varied very little between runs (SD 0.1%) and conditions (SD 0.3%). The invalid trials were omitted from the subsequent analyses.

We calculated four parameters for each movement classified as correct: movement amplitude, movement extent error, total movement time, and reaction time. Reaction time was the duration between the appearance of the target and the movement onset detection. Total movement time was defined as the duration between movement onset and the return to the starting position. Movement amplitude was defined as the difference between minimal finger extension and the starting position (almost fully extended finger). Extent error was defined as the distance between the amplitude of the exerted movement and the target position.

The data was fitted to a linear model and the distribution of residuals was assessed for normality using the Shapiro-Wilk test. We then selected an appropriate parametric (normally distributed data, one-way repeated-measures ANOVA, or paired t-test) or an equivalent non-parametric test (Friedman test or Wilcoxon signed-rank test) for comparing the means of the within-subject factor *Condition* which separated the four visual feedback types. We tested for sphericity with Mauchly’s test and applied the Greenhouse-Geisser correction to the p-values and degrees of freedom, if sphericity was detected. In addition, the sphericity adjusted p-values were corrected for the multiple tested parameters using Bonferroni’s method to limit overall type I error probability to p < 0.05. If a significant effect was found for a movement parameter, subsequently Bonferroni corrected one-sided paired t-tests (normally distributed data) or Shapiro-Wilk tests (non-normal data) were performed for multiple comparison analyses.

We also tested whether behavioural differences between conditions could be explained by correlations between the movement parameters. Correlations were assessed statistically using Pearson’s correlation coefficient. P-values were Bonferroni corrected for the multitude of analyses performed. Correlations between movement parameters were only assessed for previously found conditional differences to avoid lowering statistical power.

Finally, in a control analysis we tested whether the effects of factor *Condition* on reaction time were due to differences related to the onset of visual feedback at the beginning of a movement block. To test this, a reduced dataset which contained only trials two to nine of each experimental block was used and analysed for effects of factor *Condition* on reaction time.

### Eye data analysis

We used the Eyelink Host PC software to record eye movements (SR-Research Ltd., ON, Canada). The software automatically detected eye saccades, fixations and blinks. The data was pre-processed using Matlab [9] as well as summarised and statistically analysed using R [10]. As it was assumed that subjects would fixate the screen-centred blue cross during rest periods, eye movement traces were aligned to this position by subtracting an offset calculated separately for every individual participants and experimental run as the median eye position of the last five seconds of all rest periods. Subsequently ten eye movement parameters were calculated for every trial in both action and observation conditions: number of saccades, average duration of saccades, number of fixations, average duration of fixations, number of blinks, average duration of blinks, horizontal gaze amplitude, vertical gaze amplitude, median horizontal gaze velocity, and median vertical gaze velocity. We fitted a linear model to the data and if the residuals were found to be non-normal by Shapiro-Wilk tests we investigated differences for factors *Condition* and *Action—Observation* with non-parametric Friedman tests and Wilcoxon signed-rank tests. Otherwise two-way repeated-measures ANOVAs and t-tests were used. P-values were Bonferroni corrected for the multiple tests performed.

## Results

### Finger Movements

Overall, participants performed very similar movements for all of the four visual feedback types (Table 1). We tested for differences in the factor *Condition* on the four movement parameters with a linear model; here we only report significant results. The residuals of the linear model fit were all normally distributed as assessed by Shapiro-Wilk tests. Thus, one-way repeated measures ANOVAs and one-sided t-tests were used to test for differences.

**Table 1:** Summary of selected movement parameters.

Condition	Total movement time (ms)	Movement amplitude (%)	Movement extent error (%)	Reaction time (ms)
cursor	687 ± 26	59 ± 0.4	4 ± 0.4	395 ± 7
point light	686 ± 25	60 ± 0.4	4 ± 0.4	386 ± 9
cartoon hand	659 ± 24	65 ± 0.5	10 ± 0.6	362 ± 8
virtual hand	648 ± 24	66 ± 0.6	11 ± 0.7	372 ± 7

Mean ± standard deviations of total movement time, movement amplitude, movement extent error and reaction time for the four movement conditions. Movement amplitude is the minimum finger extension and movement extent error is movement amplitude relative to target location.

A significant difference in movement amplitude was found for factor *Condition* in a one-way repeated measures ANOVA (F_(3,75)_ = 120.39, p < 0.001). Movement amplitude was larger in the two hand feedback conditions than in the two circle conditions (virtual hand > point light, t = 11.05, p < 0.001; virtual hand > cursor, t = 12.41, p < 0.001; cartoon hand > point light, t = 14.49, p < 0.001; cartoon hand > cursor, t = 15.31, p < 0.001). Movement extent error was also significantly influenced by factor *Condition* (F_(3,75)_ = 131.13, p < 0.001). As for amplitude, movement extent error was larger in the two hand conditions than in the two circle conditions (virtual hand > point light, t = 11.71, p < 0.001; virtual hand > cursor, t = 12.67, p < 0.001; cartoon hand > point light, t = 14.52, p < 0.001; cartoon hand > cursor, t = 15.64, p < 0.001).

**Fig 2:**
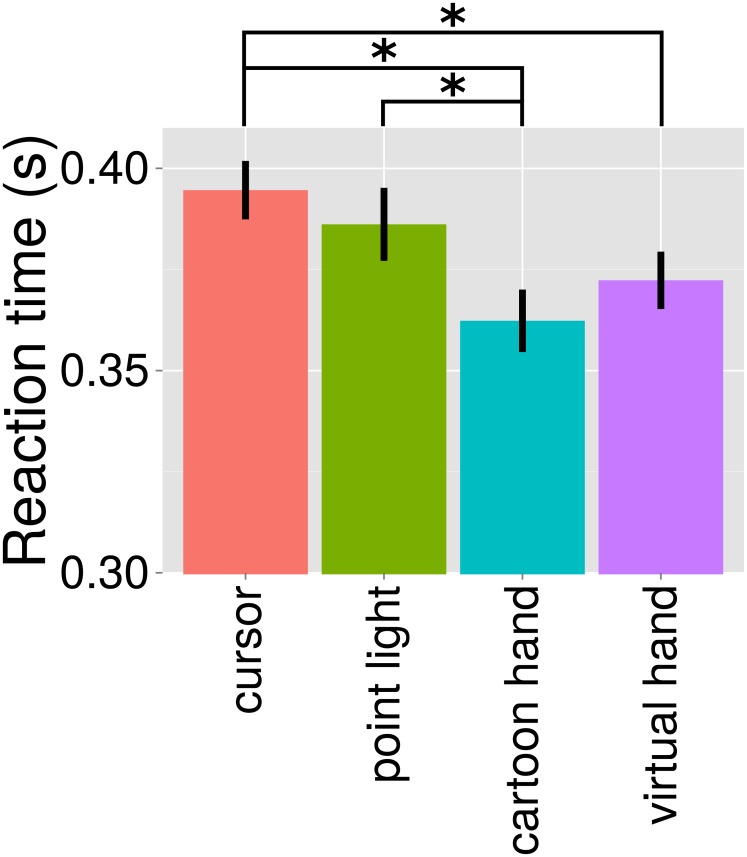
Reaction time and standard error of the four types of visual feedback. Cursor (red), point light (green), cartoon hand (cyan), and virtual hand (violet) conditions. Asterisks illustrate significant differences between conditions (p < 0.05).

No significant effect of *Condition* was found in total movement time. Instead, factor *Condition* significantly affected reaction time, (Fig 2; F_(3,75)_ = 8.87, p < 0.001). The reaction time was significantly shorter for cartoon hand than for cursor and point light conditions (point light > cartoon hand, t = 3.58, p = 0.009; cursor > cartoon hand, t = 5.04, p < 0.001). Reaction time was also significantly shorter for virtual hand compared to cursor (cursor > virtual hand, t = 3.41, p = 0.013).

Correlation analyses revealed significant correlations between movement amplitude and movement extent error but not between any of the other parameters. Movement amplitude differences were positively correlated with movement extent error differences between virtual hand and point light (r(24) = 0.93, p < 0.001), virtual hand and cursor (r(24) = 0.95, p < 0.001), cartoon hand and point light (r(24) = 0.88, p < 0.001) and between cartoon hand and cursor (r(24) = 0.92, p < 0.001).

To test if the reaction time difference depended on the visual appearance of the hand at the beginning of a block, we tested for factor *Condition* on reaction time in a reduced dataset removing the first movement of each block. Reaction time still significantly differed for factor *Condition* (F_(3,75)_ = 6.92, p = 0.001) and was significantly shorter for cartoon hand compared to cursor and point light conditions (point light > cartoon hand, t = 3.14, p = 0.026; cursor > cartoon hand, t = 4.32, p = 0.001). Reaction time was also significantly shorter for virtual hand than for cursor (cursor > virtual hand, t = 3.36, p < 0.015).

### Eye Movements

We also investigated the effect of feedback type on the participant’s eye movement trajectories. In general, the mean position of the subjects’ tracked eye stayed close to the endpoint of the controlled effector (circle or index finger) in all conditions. We investigated differences in eye movements with oculomotor parameters acquired from the eye tracking trace.

**Fig 3:**
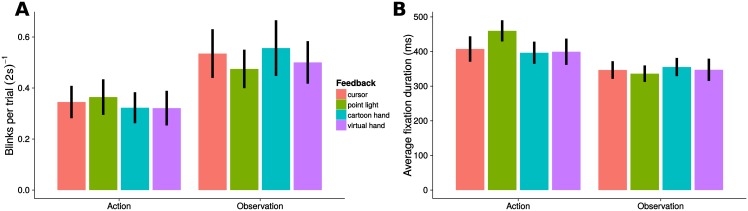
Blink rate and average duration of fixation for the four movement and the four observation conditions. Mean and standard error of **A** number of blinks per trial and **B** duration of fixation. Cursor (red), point light (green), cartoon hand (cyan), and virtual hand (violet) visual feedback.

Our primary analysis was then to test for differences between visual feedback types (factor *Condition*) in oculomotor parameters. No differences were found for factor *Condition* for any of the action or observation oculomotor parameters (Fig 3 and Table 2). In an additional analysis, we tested for differences between action and observaiton conditions (factor *Action—Observation*) and found significant differences in the number of blinks (Fig 3), fixations, and saccades per trial (Table 2). Post-hoc analyses with Wilcoxon signed-rank tests revealed that observation conditions evoked a greater number of blinks (V = 350, p < 0.001), fixations (V = 318, p < 0.001), and saccades (V = 296, p = 0.001) than action conditions. Next, we tested for differences in the average durations of fixations (Fig 3), blinks, and saccades. Factor *Action—Observation* was significant for all the three measures (Table 2). The duration of fixations was significantly smaller for observation than for action conditions (t = −5.04, p < 0.001, t-test). Instead, the durations of blinks (V = 314, p < 0.001) and of saccades (V = 306, p < 0.001) were significantly greater in observation conditions than in action conditions as revealed by Wilcoxon signed-rank tests. No significant effects were found for the other oculomotor parameters (Table 2).

**Table 2:** Analyses of oculomotor parameters.

Parameter	Action				Act.–Obs.	Observation			
	cursor	p. light	c. hand	v. hand	p	cursor	p. light	c. hand	v. hand
Sac.(2*s*^−1^)	3.7 ± 3.7	3.7 ± 3.7	3.6 ± 3.6	3.7 ± 3.7	0.017*	4.2 ± 4.2	3.9 ± 3.9	4.1 ± 4.1	4.2 ± 4.2
Fix.(2*s*^−1^)	3.0 ± 3.0	3.0 ± 3.0	2.9 ± 2.9	3.1 ± 3.1	0.004*	3.5 ± 3.5	3.3 ± 3.3	3.5 ± 3.5	3.6 ± 3.6
Bli.(2*s*^−1^)	0.3 ± 0.3	0.4 ± 0.4	0.3 ± 0.3	0.3 ± 0.3	<0.001*	0.5 ± 0.5	0.5 ± 0.5	0.6 ± 0.6	0.5 ± 0.5
ΔSac.(*ms*)	45.9 ± 45.9	49.7 ± 49.7	44.7 ± 44.7	48.5 ± 48.5	0.017*	56.3 ± 56.3	52.5 ± 52.5	57.2 ± 57.2	56.9 ± 56.9
ΔFix.(*ms*)	407.4 ± 407.4	459.8 ± 459.8	396.5 ± 396.5	399.4 ± 399.4	0.017*	346.7 ± 346.7	336.1 ± 336.1	355.3 ± 355.3	347.3 ± 347.3
ΔBli.(*ms*)	51.6 ± 51.6	51.6 ± 51.6	43.1 ± 43.1	42.8 ± 42.8	<0.001*	67.3 ± 67.3	62.1 ± 62.1	68.0 ± 68.0	71.3 ± 71.3
Δ*x*(*px*)	111.5 ± 111.5	95.6 ± 95.6	98.8 ± 98.8	106.7 ± 106.7	1.000	108.1 ± 108.1	94.0 ± 94.0	94.2 ± 94.2	102.9 ± 102.9
Δ*y*(*px*)	182.4 ± 182.4	176.8 ± 176.8	181.3 ± 181.3	181.0 ± 181.0	1.000	183.1 ± 183.1	174.2 ± 174.2	173.0 ± 173.0	180.8 ± 180.8
$\Delta v_{x}\left(\frac{px}{s}\right)$	48.3 ± 48.3	43.2 ± 43.2	43.9 ± 43.9	47.8 ± 47.8	1.000	53.1 ± 53.1	44.1 ± 44.1	45.6 ± 45.6	43.6 ± 43.6
$\Delta v_{y}\left(\frac{px}{s}\right)$	63.0 ± 63.0	61.4 ± 61.4	63.4 ± 63.4	66.9 ± 66.9	1.000	66.3 ± 66.3	61.3 ± 61.3	65.4 ± 65.4	64.3 ± 64.3

Mean ± standard deviations of oculomotor parameters. Sac.: Saccade; Fix.: Fixation; Bli.: Blink; ΔSac.: Saccade duration; ΔFix.: Fixation duration; ΔBli.: Blink duration; Δ*x*: amplitude x; Δ*y*: amplitude y; Δ*v*_*x*_: velocity x; Δ*v*_*y*_: velocity y; px: pixel; Act.–Obs.: factor *Action—Observation*; p. light: point light; c. hand: cartoon hand; v. hand: virtual hand. p-values were Bonferroni corrected.

## Discussion

In our experiment, participants initiated visually-guided finger movements faster when receiving feedback in the form of a hand than when receiving simple abstract feedback in the form of circles. This reaction time difference was significant for three out of four hand versus circle pairwise comparisons. The faster movement initiation for hand feedback was accompanied by a larger movement amplitude and a larger movement extent error. These results suggest that feedback with visual resemblance to relevant body parts changes how movements are perceived and influences how they are performed.

We analysed movement differences between feedback types in four parameters: finger movement amplitude, movement extent error, total movement time and reaction time. Subjects moved their index finger with larger amplitudes in the conditions with hand feedback compared to the conditions with circle feedback. Although we had matched the sizes of the fingertip representations (virtual and cartoon hand) with the sizes of the circles (point light and cursor), we did not know the exact visual reference point that participants used to intersect the circular targets with the virtual finger representations, possibly affecting the amplitude and accuracy results.

The observed movement extent error was slightly larger in the hand than in the circle feedback conditions, contradicting previous results suggesting more accurate movement planning and thereby also execution when viewing a hand instead of a cursor [2, 3]. However, these studies differ from our study in that they allowed vision of the hand only in the movement planning stage and in that they used an arm pointing instead of a finger reaching task. The pointing task required a high movement accuracy to correctly intersect the target, while our task only required flexion of the index finger to a certain bending angle. Furthermore, movement extent error in all of our experimental conditions was small, such that the virtual effector still largely intersected the target circle. Hence, participants did complete the task correctly in all feedback conditions and might therefore not have tried to increase accuracy. Instead, the observed difference in movement extent error might be related to the difference in movement amplitude suggested by strong correlations between the two parameters.

The most interesting result of our study was the significantly shorter reaction time for both hand feedback conditions compared to the cursor feedback condition, and for cartoon hand compared to point light. These differences were not related to differences in the other movement parameters, as the correlation analysis showed. One possible explanation for this surprising result might come from the fact that body part representations have been found to be processed and perceived differently compared to non-body part representations [12]. This phenomenon is widely accepted, although alternative theories proposed that body parts might simply be more familiar than non-body parts [13]. However, in the brain, regions exist specialized for visual processing of human body parts [14, 15]. Thus, it could be the case that visual body part information in the two hand conditions might have been processed faster than point light or cursor circles, leading to faster movement initiation.

Since our experimental task did not set a reaction time goal, it is not clear whether the difference in reaction time would also be observed in an experiment that systematically rewards fast movement initiation. However, even if this was the case, it is still interesting that movement initiation can be influenced by priming via visual feedback with body part information alone. Previous studies suggested that the joint configuration information contained in hand feedback provided the key advantages for motor control in reaching movements to varying target directions [2, 3]. Our experiment showed that it was not joint configuration information, which was also available from the point light feedback condition, but rather body part information that determined the influence of hand feedback on movement behaviour. However, this result could depend on our specific task, which did not contain requirements for movement direction and might thus not have benefited from visual joint configuration information. Despite the observed difference in reaction time, total movement time was not influenced by feedback type.

In addition to the reported differences between hand and circle feedback, we tested for differences between virtual and cartoon hand feedback and between point light and cursor feedback. Consistent with the findings from motor interference experiments [5–7], appearance and spatial information differences between virtual and cartoon hand feedback did not significantly influence behaviour. Hence, motor control did not benefit from appearance and spatial information in our task. However, more realistic virtual or real hand feedback than the one we provided might influence body part related visual processing and could thus influence behaviour. This potential effect should be investigated in future experiments. Kinematic movement parameters were also not significantly affected by the visual finger joint information provided in the comparison between point light and cursor feedback. We suggest that the mechanical constraint of the hand stabilization tube held by the other fingers may have been a key factor for providing proprioceptive joint position information resulting in this observation. Our results thus do not specifically contradict previous findings of accuracy increases in tasks particularly benefiting from visual arm configuration information [2, 3].

Measure of reaction time is a relevant component of performance in many arm and finger tasks employed in rehabilitation. Some training tasks require patients to move their upper limbs rapidly to intercept on-screen items [16, 17]. In such tasks, long reaction times result in task failure or low performance. Because of its importance for task performance, reaction time is often assessed by virtual reality systems as one of the outcome measures of rehabilitation training [18]. Furthermore, standard upper limb and finger function assessments such as the Wolf Motor function test quantify manual function by the time to complete a functional movement [19]. Even though, performance in these tests is not exclusively determined by reaction time, faster reaction times have the potential to facilitate these tasks and thereby lead to an increase of the final score for upper limb function. Hence, reaction time can influence motor function training and functional outcome measures.

The four visual feedback conditions contained images of different stimulus size, especially between the hand and the circle feedback conditions. The stimulus size might have affected attention and thus affected the observed reaction times. To test this alternative hypothesis we performed two additional control analyses. Firstly, we assessed ten oculomotor parameters, some known to be related to attention. Secondly, we tested the feedback effect on reaction time in a reduced dataset in which the first trial (containing the stimulus onset) of each block was removed.

Differences in oculomotor parameters were found between action and observation conditions, demonstrating the reliability of the chosen oculomotor parameters for detecting these attentional effects. However, we did not find effects of visual feedback type for any of the tested oculomotor parameters, suggesting that attention was not affected by visual stimulus size. In particular, blink frequency and blink duration are known to reliably indicate less attention due to fatigue [20]. As these parameters only differed between action and observation conditions, but not between feedback types, alertness was not affected by the stimulus type. Fixation duration is also known to vary with the amount of visual attention devoted to a particular stimulus in a visual search task [21]. We found that fixation duration did not differ between feedback types, thus demonstrating that visual attention was not affected by the stimulus size. Additional oculomotor parameters including the number of fixations, number of saccades and saccade duration were also only significantly affected by the difference between passive observation and active movement, and not by the feedback type.

In the control analysis with the reduced data set, we removed the first trial of each block. This was done to remove the possible effect of trials in which the stimulus appeared on the screen for the first time. We found that even with the removed first trials, the significant reaction time effect was still present. This showed that the possible effect of stimulus size during stimulus onset could not explain the reaction time difference.

### Limitations

There are some limitations to our study which should be mentioned. Firstly, our definition of movement onset could have influenced our measure of reaction time. Nevertheless, reaction time should be similarly influenced for all feedback types. Secondly, the virtual and cartoon hand stimuli activated more pixels than the point light and cursor conditions due to size and visual texture differences. However, our eye movement data and our analysis on a reduced dataset showed that it is unlikely that detection speed, alertness or visual attention were affected by the size of the visual stimulus. Future experiments might test additional visual stimuli to confirm our results. Thirdly, we assumed that input to the data glove came from index finger flexion—extension movements in a horizontal plane due to the instructions we gave. However, non planar finger adduction-abduction movements were still possible, which our data glove would not have measured. Out-of-plane movements would have conflicted with the provided visual feedback consisting of two dimensional virtual finger movements. However, it is unlikely that significant out-of-plane index finger movements occurred, due to the mechanical restraint offered by the adjacent middle finger holding the stabilization tube. Furthermore, occasional out-of-plane movements would presumably be similarly distributed across feedback types as conditions were presented in random order during the experiment.

### Conclusion

Our study highlights the importance of hand feedback for action perception, motor planning, and execution. Surprisingly, we found behavioural differences in movement initiation for observing hand compared to circle feedback during visually guided finger movements: reaction times of self-initiated movements with two slightly different types of hand feedback were shorter than reaction times of point light or cursor feedback. These findings challenge some of the assumptions implicit in many movement feedback studies that rely on simple cursor feedback for many tasks. Hand feedback might be an important part of controlling visually-guided movements and it remains to be seen to which extent neural processes are influenced by observing hand feedback versus simple cursor feedback. Our findings suggest potential benefits for the use of hand representations in improving performance in computer-mediated training and interaction systems for different applications such as rehabilitation and skill improvement.
