# Correction: Virtual Hand Feedback Reduces Reaction Time in an Interactive Finger Reaching Task

**DOI:** 10.1371/journal.pone.0176655

**Published:** 2017-04-24

**Authors:** Johannes Brand, Marco Piccirelli, Marie-Claude Hepp-Reymond, Manfred Morari, Lars Michels, Kynan Eng

The standard deviations of the oculomotor parameters in Table 2 are incorrect. Please see the corrected Table 2 here.

**Table 2:** Analyses of oculomotor parameters.

Parameter	Action				Act.–Obs.	Observation			
	cursor	p. light	c. hand	v. hand	p	cursor	p. light	c. hand	v. hand
Sac. (2*s*^*-1*^)	3.7 ± 0.4	3.7 ± 0.3	3.6 ± 0.4	3.7 ± 0.5	0.017*	4.2 ± 0.4	3.9 ± 0.3	4.1 ± 0.4	4.2 ± 0.5
Fix. (2*s*^*-1*^)	3.0 ± 0.4	3.0 ± 0.3	2.9 ± 0.4	3.1 ± 0.5	0.004*	3.5 ± 0.4	3.3 ± 0.3	3.5 ± 0.4	3.6 ± 0.5
Bli. (2*s*^*-1*^)	0.3 ± 0.1	0.4 ± 0.1	0.3 ± 0.1	0.3 ± 0.1	<0.001*	0.5 ± 0.1	0.5 ± 0.1	0.6 ± 0.1	0.5 ± 0.1
ΔSac.(*ms*)	45.9 ± 3.9	49.7 ± 4.6	44.7 ± 4.0	48.5 ± 6.9	0.017*	56.3 ± 5.9	52.5 ± 4.6	57.2 ± 6.0	56.9 ± 6.2
ΔFix.(*ms*)	407.4 ± 36.7	459.8 ± 30.6	396.5 ± 32.1	399.4 ± 37.9	0.017*	346.7 ± 25.5	336.1 ± 23.9	355.3 ± 26.4	347.3 ± 32.2
ΔBli.(*ms*)	51.6 ± 10.2	51.6 ± 10.8	43.1 ± 10.1	42.8 ± 12.2	<0.001*	67.3 ± 10.8	62.1 ± 10.3	68.0 ± 11.1	71.3 ± 12.5
Δ*x*(*px*)	111.5 ± 7.9	95.6 ± 6.7	98.8 ± 6.4	106.7 ± 6.3	1.000	108.1 ± 6.0	94.0 ± 6.6	94.2 ± 5.6	102.9 ± 5.9
Δ*y*(*px*)	182.4 ± 4.6	176.8 ± 2.1	181.3 ± 5.1	181.0 ± 4.0	1.000	183.1 ± 4.5	174.2 ± 4.2	173.0 ± 3.3	180.8 ± 3.5
$\Delta v_{x}\left(\frac{px}{s}\right)$	48.3 ± 1.9	43.2 ± 1.2	43.9 ± 1.6	47.8 ± 2.0	1.000	53.1 ± 4.6	44.1 ± 2.2	45.6 ± 1.4	43.6 ± 1.5
$\Delta v_{y}\left(\frac{px}{s}\right)$	63.0 ± 2.0	61.4 ± 1.3	63.4 ± 1.9	66.9 ± 1.9	1.000	66.3 ± 2.3	61.3 ± 2.2	65.4 ± 1.8	64.3 ± 2.1

Mean ± standard error of oculomotor parameters. Sac.: Saccade; Fix.: Fixation; Bli.: Blink; ΔSac.: Saccade duration; ΔFix.: Fixation duration; ΔBli.: Blink duration; Δx: amplitude x; Δy: amplitude y; Δ*v*_*x*_: velocity x; Δ*v*_*y*_: velocity y; px: pixel; Act.–Obs.: factor *Action–Observation*; p. light: point light; c. hand: cartoon hand; v. hand: virtual hand. p-values were Bonferroni corrected.
